# Development and validation of a m^6^A -regulated prognostic signature in lung adenocarcinoma

**DOI:** 10.3389/fonc.2022.947808

**Published:** 2022-10-11

**Authors:** Yaxin Chen, Lei Xia, Yuxuan Peng, Gang Wang, Liyun Bi, Xue Xiao, Cui Li, Weimin Li

**Affiliations:** ^1^ Institute of Respiratory Health, Frontiers Science Center for Disease-related Molecular Network, West China Hospital, Sichuan University, Chengdu, China; ^2^ Institute of Hydrobiology, Chinese Academy of Sciences, Wuhan, China; ^3^ Precision Medicine Research Center, West China Hospital, Sichuan University, Chengdu, China; ^4^ Department of Respiratory and Critical Care Medicine, West China Hospital, Sichuan University, Chengdu, China; ^5^ The Research Units of West China, Chinese Academy of Medical Sciences, West China Hospital, Chengdu, China

**Keywords:** m^6^A, prognosis, signature, lung adenocarcinoma, epitranscriptomic

## Abstract

Lung adenocarcinoma (LUAD) is the most frequent subtype of lung cancer, with a 5-year survival rate of less than 20%. N6-methyladenosine (m^6^A) is the most prevalent RNA epigenetic modification in eukaryotic cells, and post-transcriptionally regulates gene expression and function by affecting RNA metabolism. The alterations of functionally important m^6^A sites have been previously shown to play vital roles in tumor initiation and progression, but little is known about the extent to which m^6^A-regulated genes play in prognostic performance for patients with LUAD. Here, we presented an overview of the m^6^A methylome in LUAD tissues using transcriptome-wide m^6^A methylation profiles, and found that differentially methylated transcripts were significantly enriched in tumor-related processes, including immune response, angiogenesis and cell-substrate adhesion. Joint analysis of m^6^A modification and gene expression suggested that 300 genes were regulated by m^6^A. Furthermore, we developed a m^6^A-regulated prognosis-associated signature (m^6^A-PPS) by performing a multi-step process. The m^6^A-PPS model, a 15-gene set, was qualified for prognosis prediction for LUAD patients. By regrouping the patients with this model, the OS of the high-risk group was shorter than that of the low-risk group across all datasets. Importantly, patients with high m^6^A-PPS scores respond better to immunotherapeutic. Our results provide a valuable resource for understanding the important role of epitranscriptomic modifications in the pathogenesis of LUAD, and obtain potential prognostic biomarkers.

## Introduction

Lung cancer is the leading cause of cancer-related deaths in China and worldwide, and lung adenocarcinoma (LUAD) is the most frequent subtype of non-small cell lung cancer, with less than a 20% 5-year survival rate (1). Despite significant improvements in diagnosis, surgery, radiotherapy and molecular therapeutics, sustained responses are rare and prognosis remains poor (2). Tumor molecular heterogeneity among patients is one of the key factors that may contribute to worse therapeutic outcomes. Thus, combining more comprehensive molecular characterization may aid in biomarker discovery to improve the prognosis of LUAD patients.

As an important mediator in almost all the RNA cycle stages, N6-methyladenosine (m^6^A) is the most abundant internal modification in eukaryotic mRNA, and is dynamic and reversible (3). It is deposited by m^6^A writers (in particular the METTL3-METTL14 complex (4)), and erased by m^6^A erasers (FTO (5) and ALKBH5 (6)). With recognized by specific m^6^A reader proteins, m^6^A modulates different post-transcriptional processes. For example, the well-studied reader YTHDF2 recognizes m^6^A and regulates mRNA degradation (7), and YTHDC1 affects alternative splicing and nuclear export of mRNA (8). Accumulating studies have reported that alterations in functionally important m^6^A sites were correlated with the risk of malignancy of tumors at multiple levels (9). The m^6^A-mediated regulatory pathways affect different biological processes, such as drug and stress response, development and immune (10, 11). However, the roles and function of m^6^A in the development and tumorigenesis of LUAD have not been fully clarified. Furthermore, prognosis biomarkers selected from m^6^A targeting genes remain undiscovered.

Considerable evidences have shown that m^6^A-mediated expression changes in tumor-related genes are critical to cancer progression and clinical outcome (12). For example, m^6^A-mediated transcript stability leads to increased expression of lncRNA ABHD11-AS1, which promotes tumor cell proliferation and results in the poor prognosis of NSCLC patients (13). Previous studies have explored the molecular characteristics, potential functions and prognostic values of m^6^A regulators (writers, erasers and readers) and m^6^A-related lncRNAs (14–17). However, to date, comprehensive and integrated m^6^A modification and transcriptomic analyses that identify m^6^A-regulated genes as candidate prognostic markers remain scarce, and there is a lack of MeRIP-Seq data from tumor tissues. Most recently, Sun et al. (18) profiled m^6^A map of six paired tissue specimens using microarray technology and identified 10 prognostic m^6^A-regulated mRNAs in LUAD. Nevertheless, the prognostic value of m^6^A-marked mRNAs/lncRNAs for lung cancer has not been well explored.

In this study, we not only depicted the features of m^6^A methylome in LUAD by parsing transcriptome-wide m^6^A profiles, but also developed a prognostic signature based on m^6^A-regulated genes. A m^6^A-regulated prognosis-associated signature (m^6^A-PPS, a 15-gene set) had a superior ability to predict survival, and patients with high risk had shorter overall survival than those with low risk across all datasets. Furthermore, high-risk individuals could have a better response to immunotherapy. Taken together, our results demonstrated that developing a prognostic signature by fully taking advantage of m^6^A-mediated epigenetic alternations on expression is competent to improve the ability of prognostic prediction for LUAD patients.

## Materials and methods

### Collection of tissue specimens

All specimens used in the study were collected from West China Hospital of Sichuan University (Chengdu, China). Patients with primary LUAD were randomly selected from October 2019 to December 2020 and did not undergo any anti-cancer treatments before surgery. Primary tumor tissue and paired non-cancerous adjacent tissues (NATs, ≥5 cm from the tumor margin) were surgically resected, immediately preserved in RNAlater stabilization solution and then stored in a -80°C refrigerator until being used for RNA extraction. A total of 12 specimens were collected with clinical information including age, gender, pathology, tumor size and tumor stage (TNM staging according to AJCC cancer staging system 8^th^ edition), smoking status, and family history. The detailed clinical information of the patients was listed in **Supplementary Table S1**. All patient samples were obtained with the hospital’s approval of the Research Ethics Committee and all participants provided written informed consent.

### RNA extraction

Tissues were ground by using the Tissue Lyser System (Tissuelyser-24, Shanghai Jing Xin) and RNA was extracted from tissues by using the phenol–chloroform method. Samples exhibiting an RNA integrity number (RIN) greater than 6.0 were included in the study. The quality of total RNA was detected by Bioptic Qseq100 Bio-Fragment Analyzer. All samples had an RIN of 6.9 or greater. DNase I (Invitrogen™, Cat# EN0525) treatment was adopted to remove DNA contamination.

### MeRIP-seq library preparation and sequencing

m^6^A immunoprecipitation was performed by using an Epi™ m^6^A immunoprecipitation kit (Epibiotek™, Cat#R1804). Purified RNA (20μg) was fragmented into ~200 nucleotide-long fragments by incubation in magnesium RNA fragmentation buffer for 6 min at 70°C. The fragmentation was stopped by adding EDTA. Then, Zymo RNA clean and the concentrator-5 kit was used to purify fragmented total RNA (Zymo Research™, Cat# R1013). Fragmented total RNA (Input) and immunoprecipitated RNA (IP) were subjected to library construction by using Epi™ mini longRNA-seq kit (Epibiotek, Cat# E1802) according to the manufacturer’s protocols. Libraries’ quality was validated on Qseq100 Bio-Fragment Analyzer. The strand-specific libraries were sequenced on the Illumina NovaSeq 6000 platform and 150bp paired-end reads were generated.

### Collection and preprocessing of public datasets

RNA expression and relevant clinical features of The Cancer Genome Atlas (TCGA) LUAD, TCGA-LUSC and Genotype-Tissue Expression (GTEx) were downloaded from the UCSC Xena database (https://xenabrowser.net/datapages/). Gene expression was further normalized to the TPM value based on the longest transcript length and sequencing depth. The DNA copy number and somatic mutation data were downloaded from the cBioPortal database (https://www.cbioportal.org/). For microarray datasets, 6 datasets (GSE10072, GSE115002, GSE75037, GSE40791, GSE68465 and GSE72094) were downloaded from the Gene Expression Omnibus (GEO) database (https://www.ncbi.nlm.nih.gov/geo/). For datasets from platform GPL570, we downloaded raw CEL files and performed uniform process using the robust multichip average (rma) algorithm for background correction and normalization. When multiple probes correspond to the same gene symbol, the mean value was considered as the final value. Those datasets listed above were different independent studies of LUAD, among which 4 datasets (GSE10072, GSE115002, GSE75037 and GSE40791) contained paired adjacent normal samples, and another two datasets (GSE68465 and GSE72094) were employed as validation sets for the m^6^A-PPS model.

### MeRIP-seq and RNA-seq data analysis

Reads from the MeRIP-seq and RNA-seq were first filtered by removing adaptor primers and low-quality reads using Cutadapt (version 2.5). The high-quality reads were aligned to the human reference genome (GRCh38) using HISAT2 (version 2.1.0) with “–rna-strandness RF” and other default parameters. Only reads with mapping quality of more than 20 were retained using SAMtools (v1.7.0). Aligned reads were converted into bigwig format based on BPM normalization in 1 bp bin size with deepTools (v3.5.0) and visualized with Integrative Genomics Viewer (IGV). The gene expression raw counts were calculated using featureCounts (v2.0.1). TPM normalization was performed as described above. DESeq2 (v1.26.0) was utilized to identify differentially expressed genes (FDR ≤ 0.05 and | log2 (fold change) | ≥ 2).

### m^6^A peak identification and annotation

m^6^A peak calling and analysis of differential m^6^A peaks were carried out using exomePeak (v2.13.2) with default parameters. The significantly differential peaks were defined by the thresholds of *P ≤* 0.01 and |log2(fold change) | ≥1. The peak annotation and motif enrichment were analyzed by HOMER with “-len 6 -rna”. Metagene plots of m^6^A peaks and reads were created by the Bioconductor Guitar package (v1.16.0). Differentially m^6^A-methylated mRNA/ncRNA in all chromosomes (except chrX, chrY, and chrM) were displayed as a circle plot by the R package circlize (v0.4.14).

### Function enrichment analysis

GO enrichment and KEGG pathway enrichment analyses of m^6^A peaks, differential m^6^A modification and differentially expressed genes were performed using ClusterProfile (v3.6.0). FDR<0.05 indicates that the functional comment is significantly enriched.

### Construction of the risk model

Patients with LUAD (N = 462) from TCGA were randomly divided into a training set (N = 324) and a testing set (N = 138) to construct and assess the prognostic model. The glmnet R package (v4.1.2) was used to perform a least absolute shrinkage and selection operator (LASSO) Cox regression (iteration = 1000 and 10-fold cross-validation) to penalize a m^6^A-regulated-RNA prognostic signature for LUAD patients. The signature can stratify patients into high-risk and low-risk groups. The risk score calculation formula is:


$$\;Riskscore\;=\;\sum_{i=0}^{n}Coef_{i}*x_{i}$$

where Coef_i_ is the coefficient, and x_i_ is the expression value of each m^6^A-regulated RNA. The Kaplan–Meier log-rank test (survival v3.2.13), and univariate and multivariate Cox regression analyses were performed to evaluate the predictive ability of the prognostic m^6^A-PPS. Spearman’s correlation analysis was applied to evaluate the association between 23 m^6^A regulators (writers/readers/erasers) and 15 m^6^A-PPS genes using R package ggstatsplot (v0.8.0) and plots visualized with the ggcorrplot package (v0.1.3).

### Prediction of m^6^A-binding proteins

We downloaded targets of 151 RBPs from POSTAR3 (http://postar.ncrnalab.org) (19). For each RBP, the significance of overlapping between targets of the RBP and m^6^A-regulated genes identified in our study was calculated by testing whether m^6^A-regulated genes and other genes (as background) had equal fractions when identified as the targets of the RBP using chi-square tests. The q-values, an indicator of FDR, were calculated for all RBPs to screen out significant overlapping RBPs (q< 0.05).

### Exploration of m^6^A-PPS on immunotherapy response

The R package maftools v2.6.5 was used to sum and evaluate TMB score of LUAD samples (20). The TIDE score was directly downloaded from the TIDE database (http://tide.dfci.harvard.edu/) to evaluate the likelihood of the immunotherapeutic response. A higher TIDE score indicated that tumor cells were more likely to induce immune escape, thus indicating a lower response rate to immune checkpoint inhibitors treatment (21).

### Survival analysis and statistical analysis

All statistical analyses were implemented in R statistical software (v4.0.5). The time-dependent area under the ROC curve (AUC) for survival variables was conducted by the timeROC package (v0.4.0). Patients were separated into two subgroups according to the expression level of genes or risk scores, and the R package survminer (v.0.4.9) was used to determine the optimal cutoff value. The Kaplan–Meier curve of each group with the survival data was plotted using the R package survival (v3.2.13). Survival differences were measured by the log-rank test (pairwise and/or multivariate). Log rank score test was applied in Cox proportional hazards models. Statistical significance values in the figure legends correspond to p values, FDR or q values as follows: ns ≥ 0.05, *< 0.05, **< 0.01, ***< 0.0001.

## Results

### Overview of the differential m^6^A methylome in LUAD

**Figure 1:**
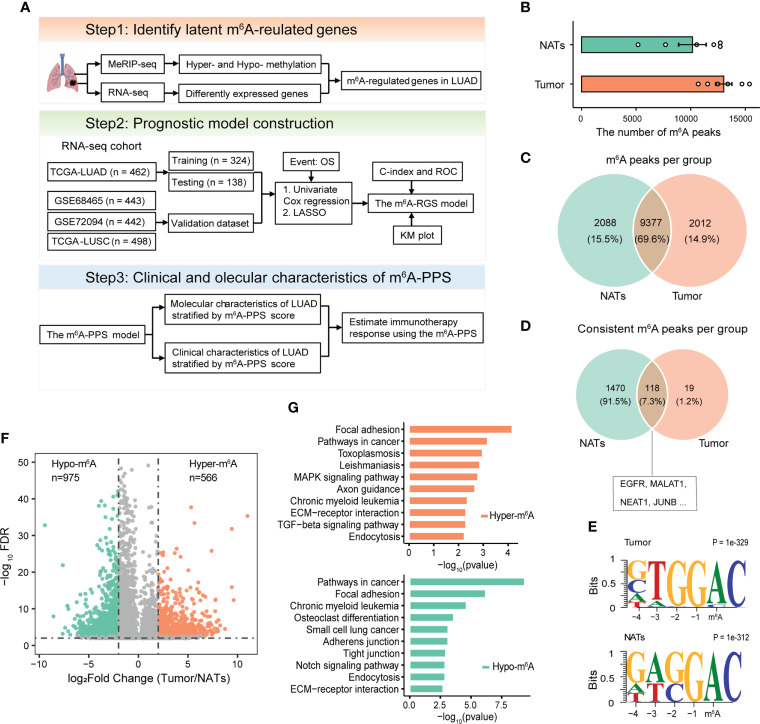
Design overview and analyses of the m^6^A methylomes of LUAD. **(A)** Schematic flow chart to identify a prognostic signature based on m^6^A-regulated genes. **(B)** The number of m^6^A peaks detected per sample in tumors and paired NATs. **(C, D)** Venn diagrams showing the overlapping m^6^A methylated transcripts **(C)** and consistent m^6^A methylated transcripts **(D)** in tumors and paired NATs. **(E)** Motif analysis for detected m^6^A peaks revealing similar consensus in tumors and paired NATs. **(F)** Volcano plot showing the differentially methylated peaks (log2FC > 1 and P-adjusted< 0.05). **(G)** KEGG pathway enrichment analysis of hyper- and hypo-methylated genes.

The overall design of this study is displayed in **Figure 1A**. To measure the topology of the m^6^A methylome in LUAD patients, six lung adenocarcinoma and paired adjacent normal fresh tissue samples collected during surgery were subjected to generate MeRIP-seq and RNA-seq data (**Figure S1A**, **Supplementary Table S1**). After quality control and filtering, the number of m^6^A peak calling per sample ranged from 5,175 to 15,383 **(** **Figure 1B** **).** Across all tissue samples per group, a total of 21,822 and 22,315 m^6^A peaks were detected in tumors and paired NATs, respectively. Of these detected peaks, approximately 70% of genes were detected as modified in both sample groups (**Figure 1C**). Furthermore, through consistent m^6^A peak calling, 154 m^6^A peaks located in 137 genes and 2,110 m^6^A peaks located in 1588 genes were detected in tumors and NATs, respectively. Some genes with m^6^A modification were shared between tumors and NATs **(** **Figure 1D**), including EGFR, a driver gene for LUAD, with enriched m^6^A modification, which is consistent with the previous finding that EGFR contains lung-specific m^6^A (22). By the unbiased motif search, m^6^A peaks were enriched for the known canonical RRACH motif (where R represents A or G; H represents A, C, or U) (**Figure 1E**). Metagene and distribution analysis of the m^6^A modification revealed that m^6^A peaks were gathered mostly in the coding sequence (CDS) and near stop codons (**Figure S1B, C**), consistent with the results of previous reports (3, 22, 23).

m^6^A aberrations are associated with the progression of cancers (24). To investigate the potential tumor specificity of the m^6^A methylome, we performed a differential m^6^A methylation analysis based on MeRIP-seq data. In total, 1,514 distinct m^6^A peaks located in the transcripts of 1,419 genes were identified, including 566 hypermethylated and 975 hypomethylated peaks (**Figures 1F**, **S1D**). Our profiling included the previously known set of m^6^A methylated sites, where > 150 transcripts were with m^6^A content in LUAD cell lines (including A549 and H1299) (**Figure S1E**). GO and KEGG pathway enrichment analyses for all m^6^A hypo- and hyper-methylated mRNA/ncRNA demonstrated that abnormal m^6^A methylation played an important role in the immune response and tumor-related processes, including positive regulation of nuclear factor-κB (NF-κB) transcription factor activity, angiogenesis and cell-substrate adhesion (**Figures 1G**, **S1F**). Taken together, these results imply that tumor-related genes are likely to be regulated by m^6^A in patients with LUAD.

### Identification of m^6^A-regulated genes in LUAD

**Figure 2:**
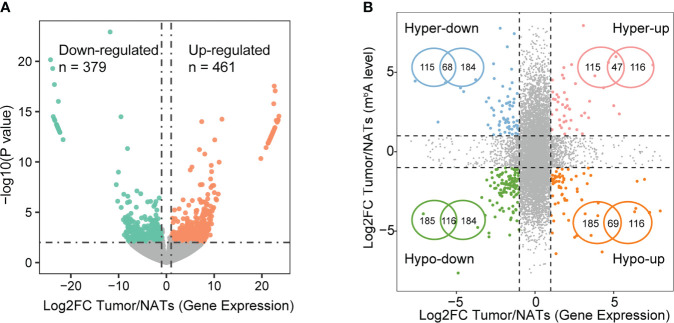
Expression alterations modulated by m^6^A signal. **(A)** Volcano plot of differentially expressed genes based on p-value< 0.01 and |log2FC| > 1. **(B)** Four-quadrant plots (left) showing genes with significant changes in both m^6^A levels and expression levels in tumors compared with NATs. Significance signals correspond to DEG and differential m^6^A methylation as follows: peaks with FDR< 0.05 and |log2FC| > 2, genes with p-value< 0.01 and |log2FC| > 1. Venn diagrams (right) showing the overlapping hyper- and hypo-methylated genes with significantly differential expression.

It is intriguing that whether abnormal m^6^A methylation affects the expression of these genes. To explore the potential function of m^6^A in regulating gene expression, m^6^A-mediated alteration of gene expression was detected regarding the MeRIP-seq input library as RNA-seq data. RNA-seq data showed that the samples from the tumor and NAT groups were separated from each other, representing a distinct expression landscape (**Figure S2A**). In total, 461 up-regulated and 379 down-regulated genes were detected in the tumor tissues (**Figure 2A**, **Supplementary Table S2**). Joint analysis of m^6^A methylation and gene expression showed 300 genes with changes in m^6^A levels and expression levels (**Figure 2B**, **Supplementary Table S3**). We defined those genes as m^6^A-regulated genes, among which 115 hypermethylated genes were significantly up-regulated (Hyper-up: 47) or down-regulated (Hyper-down: 68) and 185 hypomethylated genes were significantly differentially expressed (Hypo-up: 69 and Hypo-down: 116). Two genes with hypo-methylated (AKAP12) and the hyper-methylated (TRIM2) m^6^A peak were listed as examples and demonstrated *via* IGV tracks (**Figures S2B-D**). The m^6^A level of AKAP12 was significantly down-regulated in the tumor by 8.88-fold and conversely that of TRIM2 was significantly up-regulated by 2.38-fold.

To explore enriched regulators of m^6^A-regulated genes identified in this study, targets of 151 RBPs were enrolled. The top enriched RBP were transforming growth factor beta regulator 4 (TBRG4) and RNA splicing factor SRRM4, which are important regulators in the tumorigenic progression of several tumors (**Figure S2E**). The specific m^6^A demethylase ALKBH5 and reader YTHDF2 were also identified. Our results showed that the majority (64.4%) of m^6^A-regulated transcripts were hypo-methylated (**Figure 1F**). YTHDF2 mediates m^6^A promoting the decay of mRNA, while ALKBH5 erases m^6^A modification, which both explained the observed hypo-methylation trend in this study.

### Development of m^6^A-PPS in LUAD

**Figure 3:**
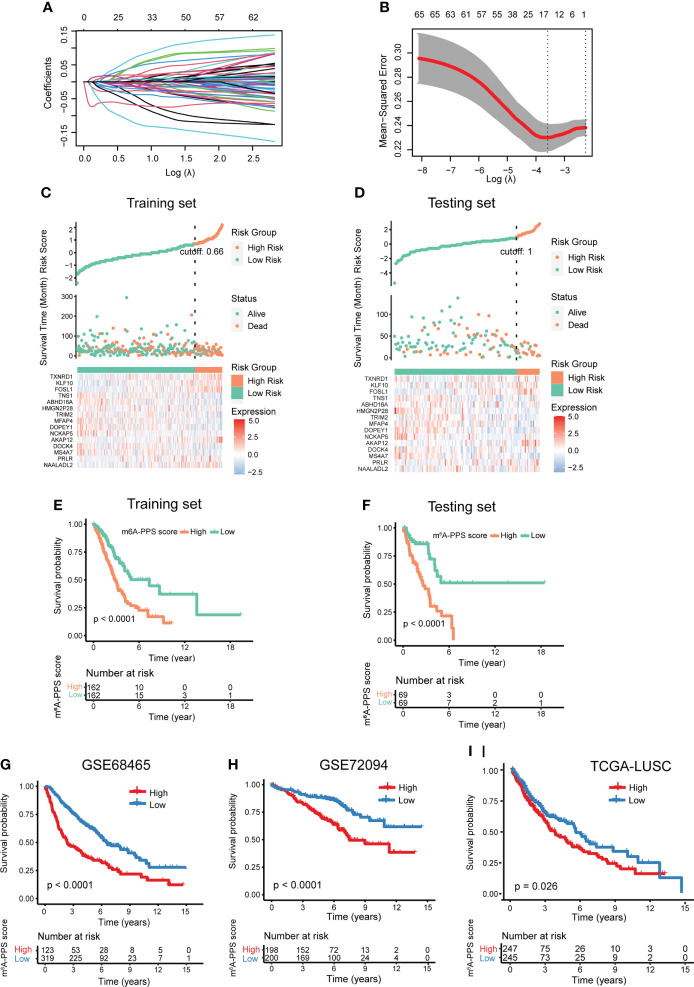
Development and validation of the m^6^A-PPS and evaluation of the prognostic value. **(A)** The tuning parameters [log(lambda)] of OS-related genes were selected to cross-verify the error curve. According to the minimal criterion and 1-se criterion, perpendicular imaginary lines were drawn at the optimal value. **(B)** The LASSO coefficient profile of 15 OS-related m^6^A-regulated genes and perpendicular imaginary lines were drawn at the value chosen by 10-fold cross-validation. **(C, D)** Distribution of risk score, survival status of LUAD patients and heatmap expression of 15 m^6^A-regulated genes in the training set **(C)** and testing set **(D)**. **(E, F)** Kaplan–Meier curves of OS for LUAD patients stratified by the median m^6^A-PPS score in the training set **(E)** and testing set **(F)**. **(G–I)** Validation of the m^6^A-PPS model in three independent cohorts.

Previous studies have reported that m^6^A regulators can be used as potential molecular markers for diagnosis and prognosis assessment, and m^6^A modification regulated by m^6^A regulators can facilitate or inhibit malignant behaviors primarily through regulating the expression of target oncogenes or tumor suppressor genes. Therefore, we next explored the clinical significance of 300 m^6^A-regulated genes in 1347 LUAD patients from three clinical cohorts (TCGA-LUAD, GSE68465 and GSE72094). A total of 462 TGCA-LUAD patients were divided into training and testing cohorts and were used to develop the prognostic signature. First, according to univariate Cox proportional hazards regression analysis, 67 m^6^A-regulated genes were significantly correlated with OS (*P*< 0.001). Next, LASSO Cox regression analysis was performed to discern the most available forecast markers and produce a prognostic indicator to predict clinical results (**Figure 3A**). A 15-gene set in the model was considered the m^6^A-regulated prognosis-associated signature (m^6^A-PPS) (**Figure 3B**). A Cox multivariate regression model was then used to calculate the Cox regression coefficient of m^6^A-PPS genes, and the m^6^A-PPS score of each patient was defined as taking the sum of the regression coefficient for each of m^6^A-PPS genes multiplied by its expression value. Detailed information on the 15 m^6^A-PPS genes was listed in **Supplementary Table S4**.

### Evaluation of the prognostic potentiality of m^6^A-PPS

To test the prognostic capability of the proposed model, patients were divided into a low m^6^A-PPS score group and a high m^6^A-PPS score group in the training, testing and entire set using the median score or optimal score as the cutoff point. ROC curve analyses were conducted to assess the prognostic accuracy of the 15 m^6^A-PPS-based classifier, with 1-, 3-, and 5-year AUCs of 0.745, 0.670 and 0.726 in training set; 0.652, 0.739 and 0.666 in testing set, respectively (**Figures S3A, B**). The calibration curve of the model demonstrated good agreement between prediction and observation in two sets (**Figures S3C, D**). We depicted the distribution of risk grades, the pattern of survival status and survival time, and expression of the m^6^A-regulated genes in the training set and testing set (**Figures 3C, D**). Kaplan–Meier survival curves demonstrated that the OS of the high m^6^A-PPS score group was shorter than that of the low m^6^A-PPS score group in the training set and testing set (**Figures 3E, F**). In addition, the m^6^A-PPS score for every individual in the entire set was calculated using the uniform formula, and there was no difference in the outcomes compared with the training set and testing set. A significant prognostic difference was observed between the two groups, where the higher m^6^A-PPS score group demonstrated worse survival than the lower group (**Figures S3E, F**). To further verify the grouping ability of the m^6^A-regulated gene model, principal component analysis (PCA) was conducted to test the difference between groups. The distributions of the high- and low-risk groups based on the entire gene expression profiles were relatively scattered (**Figure S3G**). However, the results obtained based on our model illustrated that the low- and high-risk groups had different distributions (**Figure S3H**), suggesting that the m^6^A-PPS can distinguish between the low- and high-risk groups.

To confirm the similar prognostic potentiality of the m^6^A-PPS-based classifier in different populations, three independent cohorts were employed. In GSE68465 and GSE72094 cohorts, the results of Kaplan–Meier survival analyses also revealed that the prognostic difference of LUAD patients stratified by the m^6^A-PPS score was statistically significant (**Figures 3G, H**). For the LUSC population, patients with a higher relative risk showed a trend toward higher mortality (**Figure 3I**). Taken together, the m^6^A-PPS was competent to predict the outcomes of non-small cell lung cancer.

### Independence and accuracy of the m^6^A-PPS

**Figure 4:**
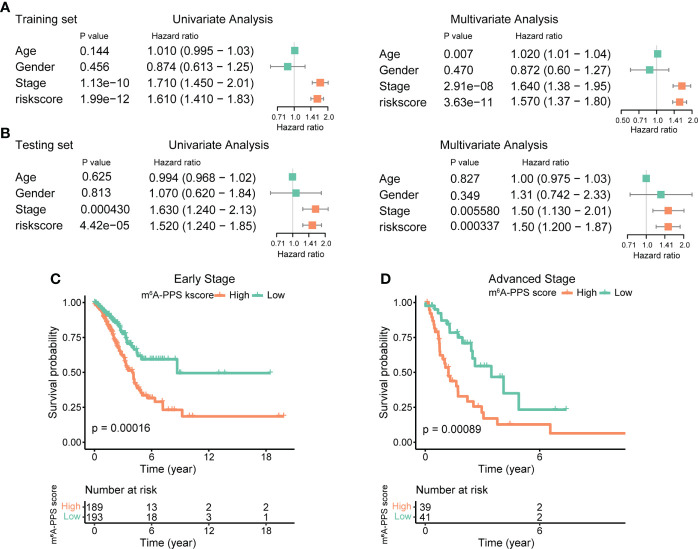
Assessment of the prognostic risk model of the m^6^A-RPS and clinical features. **(A, B)** Univariate and multivariate Cox regression analyses of correlations between the risk score for the m^6^A-RPS and clinical features in the training set **(A)** and validation set **(B)**. **(C, D)** Kaplan–Meier survival analysis of m^6^A-RPS prognostic value in stage I–II **(C)** and stage III–IV **(D)** patients in the TCGA LUAD cohort.

Univariate and multivariate analysis were performed to evaluate the prognostic value of the m^6^A-PPS and other clinicopathological characteristics in the training set and testing set. The results showed that m^6^A-PPS was significantly associated with tumor stage (Training set: HR = 1.710, 95% CI: 1.450–2.01, p< 0.001; Testing set: HR = 1.630, 95% CI: 1.240–2.130; p< 0.001) (**Figures 4A, B**), while the risk model was unrelated to age and gender. According to the subgroups classified by tumor stage, similar results were observed in early-stage (stage I-II) and advanced stage (stage III-IV) patients, and the OS of low-risk patients tended to be superior to that of high-risk individuals (**Figures 4C, D**). The nomogram comprising clinical risk features and the risk grade was fabricated to predict the one-, two- and three-year OS incidences, which suggested that the risk grade had a predominant ability in prognosis prediction, compared with clinical factors (**Figure S4**). Together, these data indicate that m^6^A-PPS was an independent prognostic factor for patients with LUAD and might be useful for clinical prognosis evaluation.

### Molecular characteristics of the m^6^A-PPS

We investigated genetic alterations of 15 m^6^A-PPS genes using somatic mutations and copy number variations (CNVs) in LUAD cohorts. NCKAP5 showed the highest mutation frequency (11%), followed by DOCK4 (5%), and the frequency of other genes was less than 3% (**Figure S5A**). For CNV, the overall average frequency of m^6^A-PPS genes was prevalently low (**Figure S5B**). We evaluated the expression of 15 m^6^A-PPS genes in normal tissues from healthy human, tumor and paired normal tissues from patients with LUAD. All m^6^A-PPS genes were differentially expressed (**Figure S5C**). When only compared with paired non-cancerous adjacent tissues in 58 LUAD patients, the expression of 12 of 15 genes was significantly differentially regulated in tumors (**Figure S6**).

**Figure 5:**
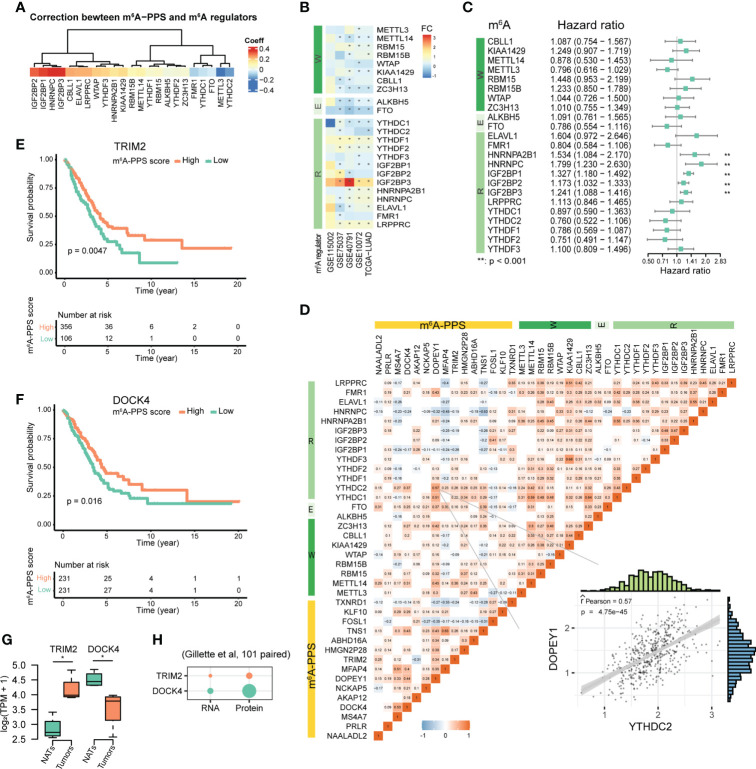
Molecular features of m^6^A-PPS and its correlation with m^6^A regulators. **(A)** Correlation between m^6^A-PPS and m^6^A regulators. **(B)** Heatmap showing the alternate expression of 23 m^6^A regulators across five independent LUAD cohorts. The asterisk represents an adjusted P value< 0.05. FC denotes log_2_Fold change between tumor vs. normal. **(C)** Forest plot of the prognostic ability of 23 m^6^A regulators. **(D)** Heatmap for the correlations between 23 m^6^A regulators and 15 prognostic m^6^A-regulated genes in the TCGA-LUAD cohort. Scatterplot showing an example of the Pearson correlation between YTHDC2 and DOPEY1 (Pearson R = 0.57). **(E, F)** OS of LUAD patients stratified by the expression levels of TRIM2 **(E)** and DOCK4 **(F)**. **(G)** Box plots showing the transcript-level differences of TRIM2 and DOCK4 in this study. **(H)** Dot plot depicting the differential expression of TRIM2 and DOCK4 at the RNA and protein levels in 101 tumors and 101 matched NATs. p-value from Wilcoxon rank-sum test (*P<0.05).

To investigate the relationship between m^6^A-PPS and 23 m^6^A regulators (including 8 writers, 2 erasers and 13 readers), we conducted Spearman correlation analysis between the mRNA expression of m^6^A regulators and the m^6^A-PPS score and between the mRNA expression of m^6^A regulators and 15 m^6^A-PPS-associated genes. The results showed that the expression of IGF2BP1/2/3 (*r* > 0.3, *P*< 0.05) and METTL3 (*r<* -0.3, *P*< 0.05) was significantly correlated with the m^6^A-PPS score (**Figure 5A**). IGF2BP3 was significantly upregulated in tumors compared with paired normal tissues across five independent cohorts and was identified as a risk factor in LUAD (HR = 1.241, *P*< 0.001) (**Figures 5B, C**). Several highly correlated relationships were observed between m^6^A regulators and m^6^A-PPS genes, among which there was a higher correlation between DOPEY1 and YTHDC2 than others (**Figure 5D**, *r* = 0.57).

The results of multivariate Cox regression analysis showed that the expression levels of TRIM2 and DOCK4 were associated with increased risk scores (**Figure S5D**), which was consistent with the results of LASSO Cox regression analysis (**Figures 3C, D**). Kaplan–Meier survival curves confirmed that higher expression of TRIM2 and DOCK4 were both prognostic for longer survival in the TCGA dataset (**Figures 5E, F**). We further verified the expression of TRIM2 and DOCK4 based on RNA-seq data from 5 LUAD patients, which suggested that TRIM2 was up-regulated and DOCK4 was down-regulated in tumor tissues compared with NATs (**Figure 5G**), which was consistent with the results from the GEPIA2 database (483 tumor and 347 normal samples) (**Figure S5E**). The Pearson correlation coefficients of the two genes and the m^6^A writer METTL14 were both more than 0.3 (*P<*0.001; TRIM2, *r* = 0.38; DOCK4, *r* = 0.31) (**Figure 5D**). Although the m^6^A levels of TRIM2 and DOCK4 were both hypo-methylation in tumors (**Supplementary Table S3**), the protein expression level of DOCK4 was increased, while TRIM2 protein expression was unchanged (**Figure 5H**), which was coherent with the fact that RNA m^6^A affects mRNA translation.

### Estimation of immunotherapy response using the m^6^A-PPS

**Figure 6:**
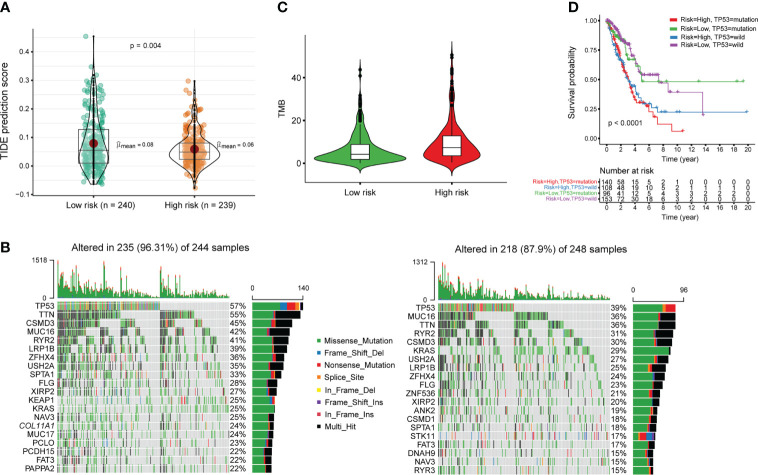
Estimation of the immunotherapy response of m^6^A-PPS in the entire TCGA cohort. **(A)** TIDE prediction difference in the high- and low-risk patients. **(B)** Oncoplot of genomic alterations of the top 20 frequently mutated genes in the high-risk group (left) and low-risk group (right). Each column corresponds to every patient. The upper bar plot shows the TMB of every individual. The right panel shows the mutation frequency and proportion of each variant type for each gene. **(C)** TMB difference in the high- and low-risk patients. **(D)** Kaplan–Meier curve analysis of OS for LUAD patients classified according to TP53 mutation status and m^6^A-PPS.

Gene Ontology (GO) enrichment analysis of tumor-enriched m^6^A modification revealed the involvement of immune-related biological processes (**Figure 1F**). Thus, we investigated the correlations between m^6^A-PPS and immunotherapeutic biomarkers. We discovered that the high m^6^A-PPS score group was more likely to respond to immunotherapy than the low m^6^A-PPS score group, indicating that m^6^A-PPS might serve as an indicator for predicting Tumor Immune Dysfunction and Exclusion (TIDE) (**Figure 6A**). Tumor mutational burden (TMB) was employed as a valid biomarker to predict the response to PD-L1 treatment (25). Based on somatic mutation data from the TGCA-LUAD cohort, the top 20 driver genes with the highest alteration frequency between the high- and low-risk subgroups were listed (**Figure 6B**). We found that the TMB scores in the high-risk group exceeded those in the low-risk group, implying that the m^6^A-PPS had a high correlation with TMB (**Figure 6C**). TP53 was used as a prognostic marker and its mutation was correlated with worse survival of LUAD patients (26). Therefore, we validated whether the m^6^A-PPS could better predict the OS outcome than TP53 mutation status (wild-type or mutated). The patients with TP53 mutation and high-risk had the worst survival outcomes compared with the other three groups (**Figure 6D**). In conclusion, patients in the high- and low-risk subgroup were distinguished more effectively in terms of the immunotherapeutic response.

## Discussion

Cancer is a heterogeneous disease, and molecular characterization of patients’ tumors becomes increasingly important to direct treatment efforts. Over the last decade, high-throughput analyses of large number of samples have significantly propelled the understanding of lung carcinogenesis, and identified biomarkers have shed light on improving patient’s outcomes. m^6^A, the most frequent modification on RNA, plays a critical role in many regulatory processes and m^6^A-mediated regulatory pathways affect disease development. Despite rapid progress, there are still noteworthy gaps in the understanding of m^6^A-regulation and function in LUAD. Here, our aim was to develop a prognostic predictor based on m^6^A-mediated genes for LUAD patients. As expected, m^6^A-PPS, a 15-gene set, had markedly better performance in prognostication than other direct gene-expression-based signatures, as well as a better response to immunotherapy, and higher TMB scores. These results implied a precise prognosis prediction approach toward patients with similar biological patterns, which was bound to achieve more reliable prognostication.

Different LUAD subtypes have distinct clinical and molecular characteristics, as well as clinical outcomes, making it almost impossible to find a therapy that fits all LUAD cases (1). Thus, many studies have focused on identifying more precise molecular signatures to predict the survival and immunotherapeutic response of LUAD patients. Molecular features, such as expression level, protein level, gene mutation or DNA methylation, provide further prognosis insights but have yet been limited to the use of single information (27, 28). m^6^A, an important regulator of gene expression, has been found to play an essential role in the prognosis, progression, and immune microenvironment of LUAD (29). However, studies on the pathological role and function of m^6^A and its target mRNAs/lncRNAs in LUAD progression remain limited, the feasibility of serving the m^6^A-regulated gene as a candidate prognostic marker in particular. Therefore, developing a m^6^A-mediated-gene-expression-based signature, as one of the main purposes of this study, is of great significance to optimize the prognostic evaluation, and m^6^A-PPS could be a novel biomarker set to guide targeted treatment for subsequent studies.

Using an analysis that correlates m^6^A effects on expression level, we identified 300 m^6^A-regulated genes, such as AKAP12 (**Figure S2**), a kinase anchor protein 12, which is critical for signal transduction and cell structure maintenance (30). However, due to the heterogeneity of tumor molecules and the small sample sizes of this study, it may also be underpowered to detect many more m^6^A-regulated genes. For potential enriched RBPs of 300 m^6^A-regulated genes, some transcription factors were identified, such as TBRG4 (**Figure S2D**), which may be a trans regulator of m^6^A in LUAD. Previous reports have shown that transcription factor-m^6^A regulator interactions may broadly exist and participate in common diseases (31).

The m^6^A-PPS consisted of 15 genes, including NAALADL2, PRLR, MS4A7, DOCK4, AKAP12, NCKAP5, DOPEY1, MFAP4, TRIM2, HMGN2P28, ABHD16A, TNS1, FOSL1, KLF10 and TXNRD1, which is not in line with previous works that reported 10 prognostic m^6^A-regulated genes (RFXAP, KHDRBS2, MAPRE3, TXN, EGFR, MGST3, USP1, ARHGEF4, CDHR5, and ADAMTS6) (18). A possible explanation for this apparent discrepancy is that m^6^A methylation is dynamic, and m^6^A motifs are methylated depending on diverse conditions. For example, for pathological stage, LUAD patients in this study stemmed from early stage, not advanced stage, and the expression level of cis and trans regulators of m^6^A and related-regulation network were variable range from in different stages. Another example is genetic determinants of m^6^A methylation (32). HMGN2P28, one of 15-gene sets, is a processed pseudogene and its expression was significantly lower in tumors than NATs in this study, but was higher in special groups (**Figure S5**). Zhao et al. reported that the m^6^A-modified pseudogene HSPA7 was identified as a novel prognostic risk factor and immunotherapy target for glioblastoma patients (33). These processed pseudogenes have evolved surveillance mechanisms through m^6^A modification instead of nonsense-mediated decay, keeping necessary pseudogene transcripts to interfere with the regulatory network of protein-coding genes (34).

Almost every stage of RNA metabolism, such as stability and translation efficiency, is regulated by m^6^A (35). In this study, most of the genes with altered m^6^A methylation levels were not accompanied by changes in expression levels, which may be because m^6^A modification of transcripts not only regulates expression levels but also influences chromatin state, RNA structure, and its interactions with specific RNA binding proteins (36, 37). Among 15 m^6^A-PPS genes, TRIM2 and DOCK4 exhibited independent prognostic value. TRIM2 (tripartite motif-containing 2), a member of the TRIM superfamily, is involved in a variety of physiological processes, including DNA repair, cell proliferation and pluripotency, transcription and signal transduction, and is also associated with carcinogenic effects in several malignancies, such as lung cancer (38) and pancreatic cancer (39). DOCK4 (dedicator of cytokinesis 4), a member of the CDM gene family encoding regulators of small GTPases, is mainly involved in maintaining cellular homeostasis (40). Mutations or reduced expression of DOCK4 can lead to lung malignancies as well as solid tumor metastasis (41). We found that although both TRIM2 and DOCK4 were hypermethylated, the expression level of TRIM2 was increased, while that of DOCK4 was decreased. The results suggested that m^6^A methylation affected on gene expression, but the regulatory function of m^6^A in mRNA remained to be fully explored.

As a decisive factor of the prognosis, different pathological stages tend to exhibit different clinical outcomes in LUAD patients. Unfortunately, patients with LUAD at the same stage could have different clinical outcomes. The established m^6^A-PPS model, based on m^6^A-regulated genes, not m^6^A-related genes, outputs a more accurate prediction for prognosis in LUAD patients. The results also provide insights for future studies on the process and mechanism of m^6^A modification of mRNAs. In this study there are shortcomings and limitations, especially the small number of specimens used, which may affect the screening of candidate m^6^A-regulated genes. Additionally, external independent datasets would be employed to validate the prediction model and the biological mechanism of m^6^A-PPS would be elucidated. Nevertheless, our results can still provide novel information for prognosis using m^6^A-PPS, thereby providing insights into their potential roles in LUAD tumorigenesis and progression.

## Conclusion

In this study, we developed and validated a m^6^A-regulated prognosis-associated signature (m^6^A-PPS) for LUAD. The 15-gene signature was qualified for prognosis prediction for LUAD patients as well as predicted response to immunotherapeutic.

## Data availability statement

The data presented in this study have been deposited in the Genome Sequence Archive (GSA) in BIG Data Center, Beijing Institute of Genomics (BIG) under accession number HRA003044 (https://ngdc.cncb.ac.cn/gsa-human/browse/HRA003044).

## Ethics statement

The studies involving human participants were reviewed and approved by The study was approved by the Ethics Committee of West China Hospital of Sichuan University (Ethics: Project identification code: 2021.292). The patients/participants provided their written informed consent to participate in this study.

## Author contributions

WL and YC conceived and designed the project, performed bioinformatic analyses and drafted and edited the manuscript with all authors providing feedback. LX and YP helped with data analysis and revised the paper. CL conducted experiments. XX collected human clinical samples. GW and LB contributed to interpreting the results. All authors contributed to the article and approved the submitted version.

## Funding

This study was supported by National Natural Science Foundation of China (Nos. 81871890, 91859203 to WL), Post-Doctor Research Project, West China Hospital, Sichuan University (2021HXBH051), Sichuan Science and Technology Support Project (No. 2022NSFSC1516 to YC) and the Fundamental Research Funds for the Central Universities (No. 2022SCU12047 to YC).

## Conflict of interest

The authors declare that the research was conducted in the absence of any commercial or financial relationships that could be construed as a potential conflict of interest.

## Publisher’s note

All claims expressed in this article are solely those of the authors and do not necessarily represent those of their affiliated organizations, or those of the publisher, the editors and the reviewers. Any product that may be evaluated in this article, or claim that may be made by its manufacturer, is not guaranteed or endorsed by the publisher.

## Supplementary material

Supplementary Figure 1 Landscape of the m^6^A methylome in LUAD patients. **(A)** Overview of the collected specimens, the corresponding clinical features of donors and high-throughput sequencing of tissues. **(B)** Metagene distribution of the enriched m^6^A peaks in tumor tissue and paired NATs. Each segment was normalized according to its average length in Refseq annotation. **(C)** Pie charts showing the distribution of enriched m^6^A peaks in tumors and paired NATs. **(D)** Circle plot showing differential m^6^A modification peaks. The statistical significance of the difference between tumor and paired NATs was determined based on log_2_FC > 1 and P-adjusted< 0.05. The first track indicates altered m^6^A peaks and the second track indicates the density distribution of altered m^6^A peaks. **(E) **Venn diagrams showing the overlapping m^6^A methylated genes among A549, H1299 and LUAD tissues. **(F)** Biology process enrichment analysis of hyper- and hypo-methylated genes.

Supplementary Figure 2 Representative IGV views of m^6^A signals and identification of enriched RBPs of m^6^A-regulated genes. **(A–C)** Tracks showing the read coverage of the IPs and inputs of the representative individuals as well as highlighted m^6^A peaks on EGFR, AKAP12 and TRIM2. The peaks are located in the 3’UTR of EGFR **(A)** and the exon regions of AKAP12 **(B)** and TRIM2 **(C)**. The tracks are shown for optimal viewing. **(D)** Bubble chart presenting enriched regulators of m^6^A-regulated genes detected in this study.

Supplementary Figure 3 Evaluation of the prognostic value of a 15-gene set. **(A, B)** Time-dependent ROC analysis for the prognosis prediction of the signature of OS in the training set **(A)** and testing set **(B)** and the area under curve (AUC) was calculated. **(C, D)** Calibration curve of the nomogram in the training set **(C)** and testing set **(D)**. **(E, F)** Distribution of risk score, survival status of LUAD patients and heatmap expression of 15 m^6^A-regulated genes in the entire set (E). Kaplan–Meier survival curves of LUAD patients with high- and low-risk scores in the entire set **(F)**. **(G, H)** Principal component analysis between the high- and low-risk groups based on the entire gene expression profiles **(G)** and 15 m^6^A-regulated genes **(H)**.

Supplementary Figure 4 A nomogram predicting the probability of the one-, two- and three-year overall survival.

Supplementary Figure 5 The landscape of genetic and expression alterations of m^6^A-PPS. **(A)** A total of 155 of 567 LUAD patients experienced genetic alterations of 15 m^6^A-PPS genes. The number on the right indicates the mutation frequency in each gene, and each column represents every individual. **(B)** The CNV frequency of m^6^A-PPS was low. **(C)** Average expression of 15 m^6^A-PPS genes in normal tissues from the GTEx database, tumor tissues and paired normal tissues from the TCGA-LUAD cohort. Asterisks show the level of significance among different tissue types based on the Kruskal–Wallis test (**P< 0.01, *** P< 0.001, ****P< 0.0001). **(D)** Forest plot of the prognostic ability of 15 m^6^A-regulated genes using the multivariate Cox proportional hazard model. **(E)** The mRNA expression levels of TRIM2 and DOCK4 in 483 tumors and 347 normal tissues from the GEPIA2 database.

Supplementary Figure 6 The expression overview of 15 m^6^A-PPS genes between tumor and paired normal tissues. (ns P>0.05, **P< 0.01, *** P< 0.001, ****P< 0.0001).

## Abbreviations

AUC, area under the curve; DEGs, Differentially expressed genes; GO, Gene ontology; IGV, Integrative genomics viewer; LUAD, Lung adenocarcinoma; LUSC, lung squamous cell carcinoma; LASSO, Least absolute shrinkage and selection operator; MeRIP-seq, Methylated-RNA immunoprecipitation sequencing; m^6^A-PPS, m^6^A-regulated-gene signature; NSCLC, non-small cell lung cancer; NATs, non-cancerous adjacent tissues; OS, Overall survival; PCA, Principal component analysis; RBP, RNA binding protein; ROC, Receiver operating characteristic; TCGA, The Cancer Genome Atlas; TCGA-LUAD, TCGA lung adenocarcinoma; TMB, Tumor mutational burden; TIDE, Tumor immune dysfunction and exclusion; TNM, Tumor node metastasis.
