# Corrigendum: Development and validation of a m^6^A -regulated prognostic signature in lung adenocarcinoma

**DOI:** 10.3389/fonc.2023.1309950

**Published:** 2023-11-07

**Authors:** Yaxin Chen, Lei Xia, Yuxuan Peng, Gang Wang, Liyun Bi, Xue Xiao, Cui Li, Weimin Li

**Affiliations:** ^1^ Institute of Respiratory Health, Frontiers Science Center for Disease-related Molecular Network, West China Hospital, Sichuan University, Chengdu, China; ^2^ Institute of Hydrobiology, Chinese Academy of Sciences, Wuhan, China; ^3^ Precision Medicine Research Center, West China Hospital, Sichuan University, Chengdu, China; ^4^ Department of Respiratory and Critical Care Medicine, West China Hospital, Sichuan University, Chengdu, China; ^5^ The Research Units of West China, Chinese Academy of Medical Sciences, West China Hospital, Chengdu, China

**Keywords:** m^6^A, prognosis, signature, lung adenocarcinoma, epitranscriptomic

In the published article, there was an error in affiliation **1**. Instead of **Frontiers Science Center for Disease-related Molecular Network, Sichuan University, Chengdu, China**, it should be **Institute of Respiratory Health, Frontiers Science Center for Disease-related Molecular Network, West China Hospital, Sichuan University, Chengdu, China**.

The authors apologize for this error and state that this does not change the scientific conclusions of the article in any way. The original article has been updated.

Additionally, in the published article, there was an error in the Data Availability statement. Instead of **The data presented in this study have been deposited in the Genome Sequence Archive (GSA) in BIG Data Center, Beijing Institute of Genomics (BIG) under accession number HRA003044 (** https://ngdc.cncb.ac.cn/gsa-human/s/epL1lQWd **).**

The correct Data Availability Statement appears below:

**The data presented in this study have been deposited in the Genome Sequence Archive (GSA) in BIG Data Center, Beijing Institute of Genomics (BIG) under accession number HRA003044 (** https://ngdc.cncb.ac.cn/gsa-human/browse/HRA003044 **).**

The authors apologize for this error and state that this does not change the scientific conclusions of the article in any way. The original article has been updated.

Furthermore, in the published article, there was an error in the Funding statement. **The funding statement for the Fundamental Research Funds for the Central Universities was displayed as “20826041F4147**. The correct statement is “**Fundamental Research Funds for the Central Universities (No. 2022SCU12047 to Y. Chen)**’’. The correct Funding statement appears below.

“This study was supported by National Natural Science Foundation of China (Nos. 81871890, 91859203 to WL), Post-Doctor Research Project, West China Hospital, Sichuan University (2021HXBH051), Sichuan Science and Technology Support Project (No. 2022NSFSC1516 to YC) and the Fundamental Research Funds for the Central Universities (No. 2022SCU12047 to Y. Chen)”.

The authors apologize for this error and state that this does not change the scientific conclusions of the article in any way. The original article has been updated.

