# Supplementary figures and images for: Development and validation of a m6A -regulated prognostic signature in lung adenocarcinoma
Source: Front Oncol. 2022 Oct 11;12:947808. doi: 10.3389/fonc.2022.947808 (PMC9593055; doi:10.3389/fonc.2022.947808)

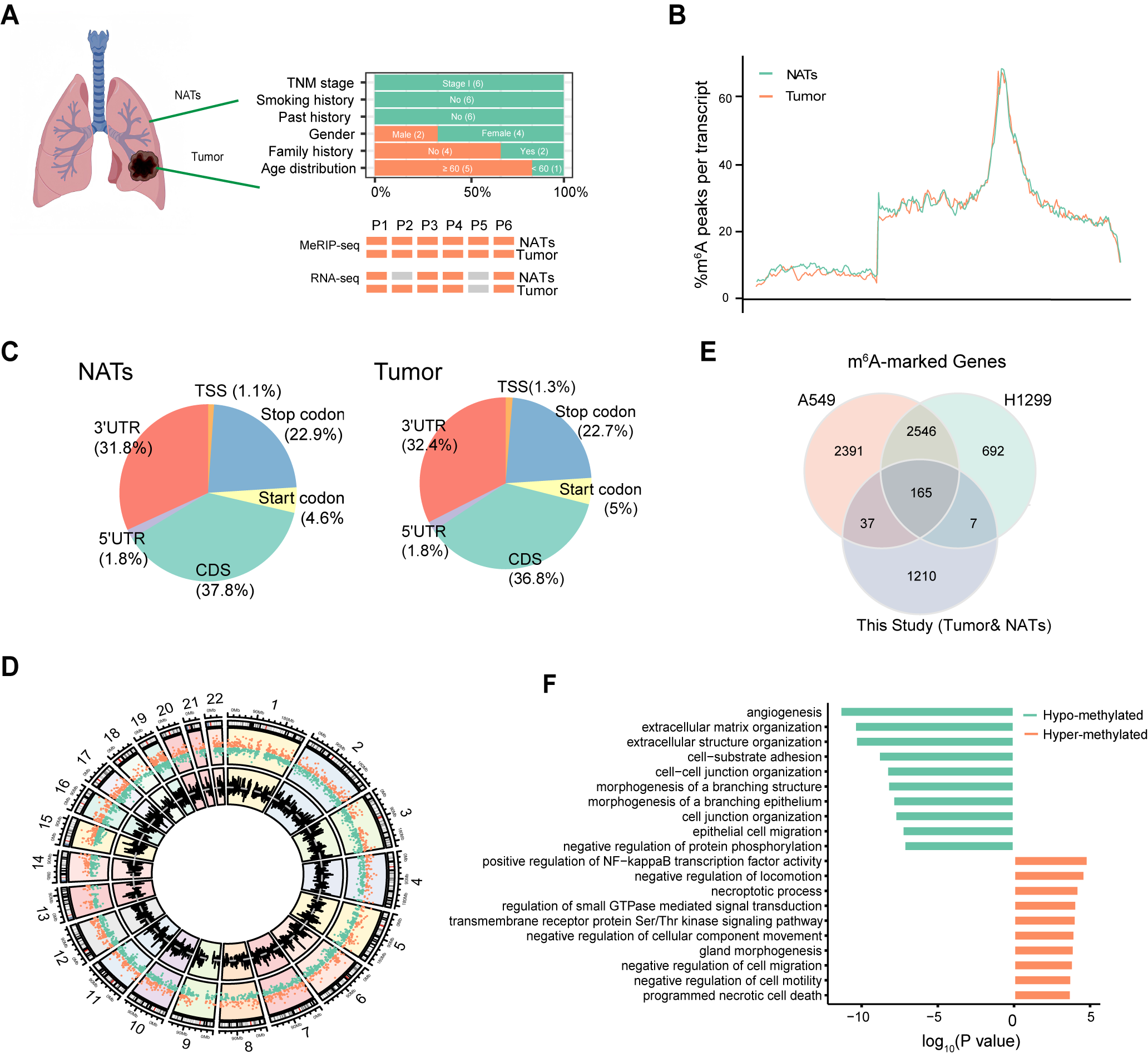

Supplement: Supplementary Figure 1 — Landscape of the m6A methylome in LUAD patients. (A) Overview of the collected specimens, the corresponding clinical features of donors and high-throughput sequencing of tissues. (B) Metagene distribution of the enriched m6A peaks in tumor tissue and paired NATs. Each segment was normalized according to its average length in Refseq annotation. (C) Pie charts showing the distribution of enriched m6A peaks in tumors and paired NATs. (D) Circle plot showing differential m6A modification peaks. The statistical significance of the difference between tumor and paired NATs was determined based on log2FC > 1 and P-adjusted< 0.05. The first track indicates altered m6A peaks and the second track indicates the density distribution of altered m6A peaks. (E) Venn diagrams showing the overlapping m6A methylated genes among A549, H1299 and LUAD tissues. (F) Biology process enrichment analysis of hyper- and hypo-methylated genes. [file Image_1.tif]

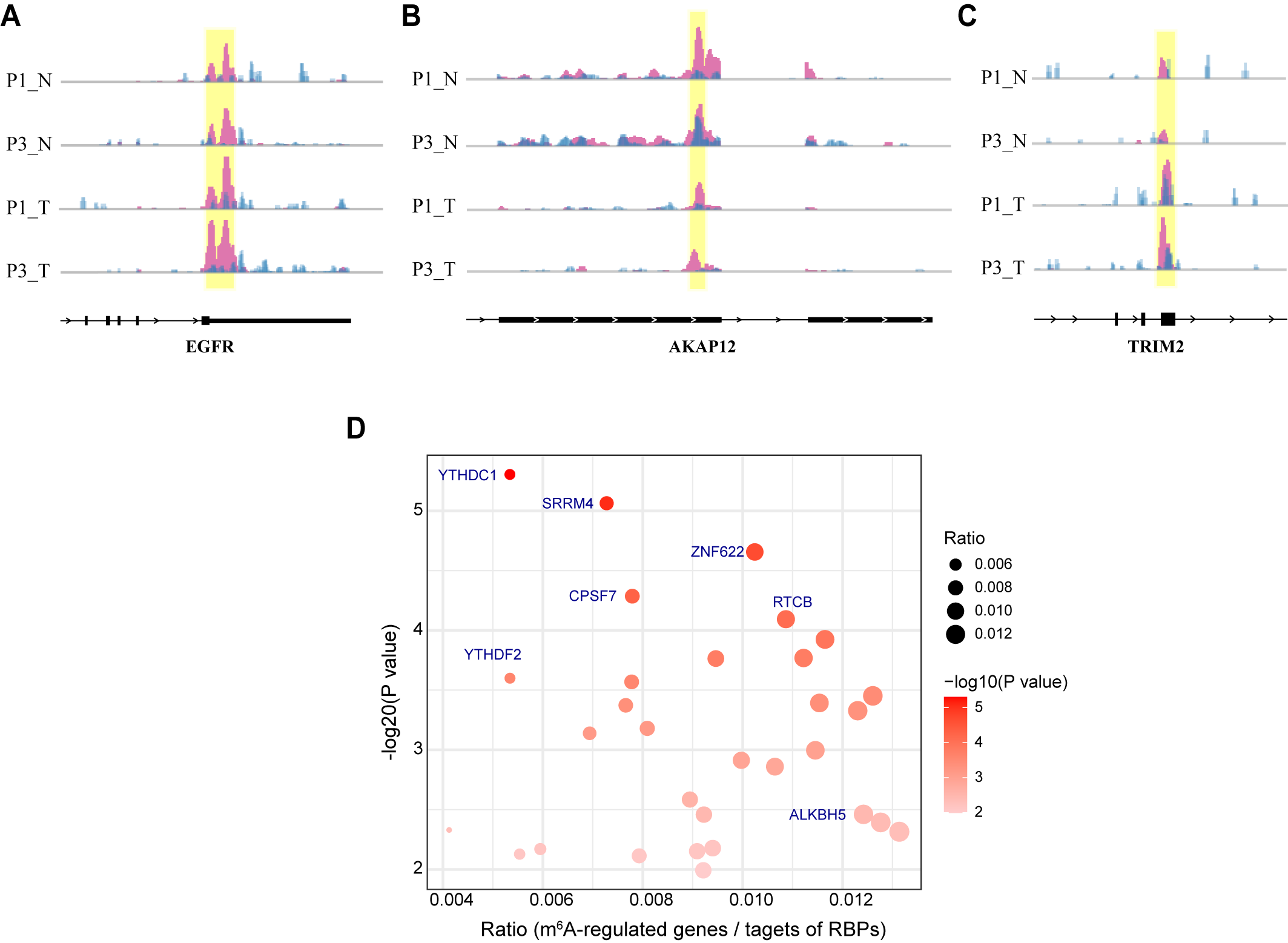

Supplement: Supplementary Figure 2 — Representative IGV views of m6A signals and identification of enriched RBPs of m6A-regulated genes. (A–C) Tracks showing the read coverage of the IPs and inputs of the representative individuals as well as highlighted m6A peaks on EGFR, AKAP12 and TRIM2. The peaks are located in the 3’UTR of EGFR (A) and the exon regions of AKAP12 (B) and TRIM2 (C). The tracks are shown for optimal viewing. (D) Bubble chart presenting enriched regulators of m6A-regulated genes detected in this study. [file Image_2.tif]

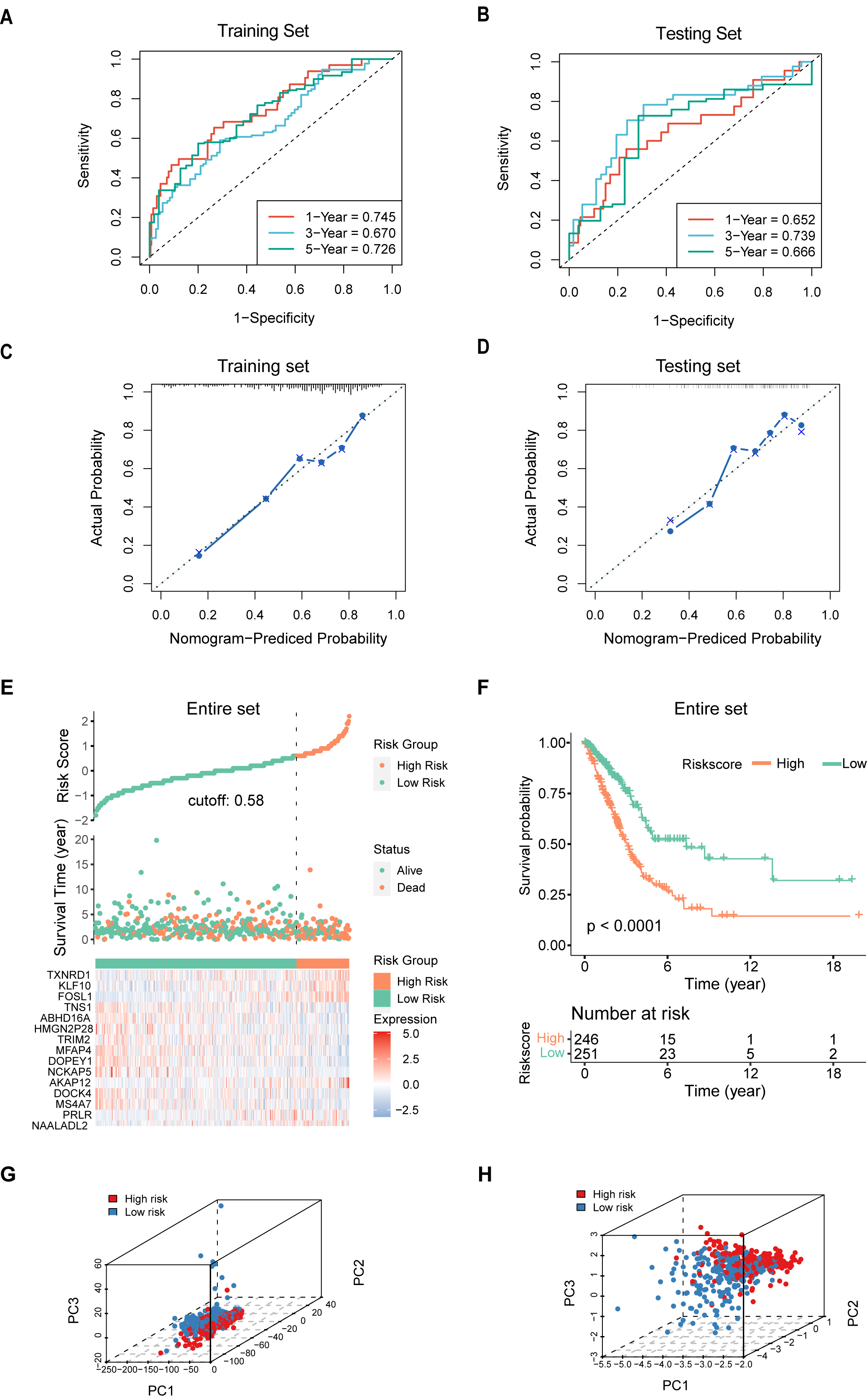

Supplement: Supplementary Figure 3 — Evaluation of the prognostic value of a 15-gene set. (A, B) Time-dependent ROC analysis for the prognosis prediction of the signature of OS in the training set (A) and testing set (B) and the area under curve (AUC) was calculated. (C, D) Calibration curve of the nomogram in the training set (C) and testing set (D). (E, F) Distribution of risk score, survival status of LUAD patients and heatmap expression of 15 m6A-regulated genes in the entire set (E). Kaplan–Meier survival curves of LUAD patients with high- and low-risk scores in the entire set (F). (G, H) Principal component analysis between the high- and low-risk groups based on the entire gene expression profiles (G) and 15 m6A-regulated genes (H). [file Image_3.tif]

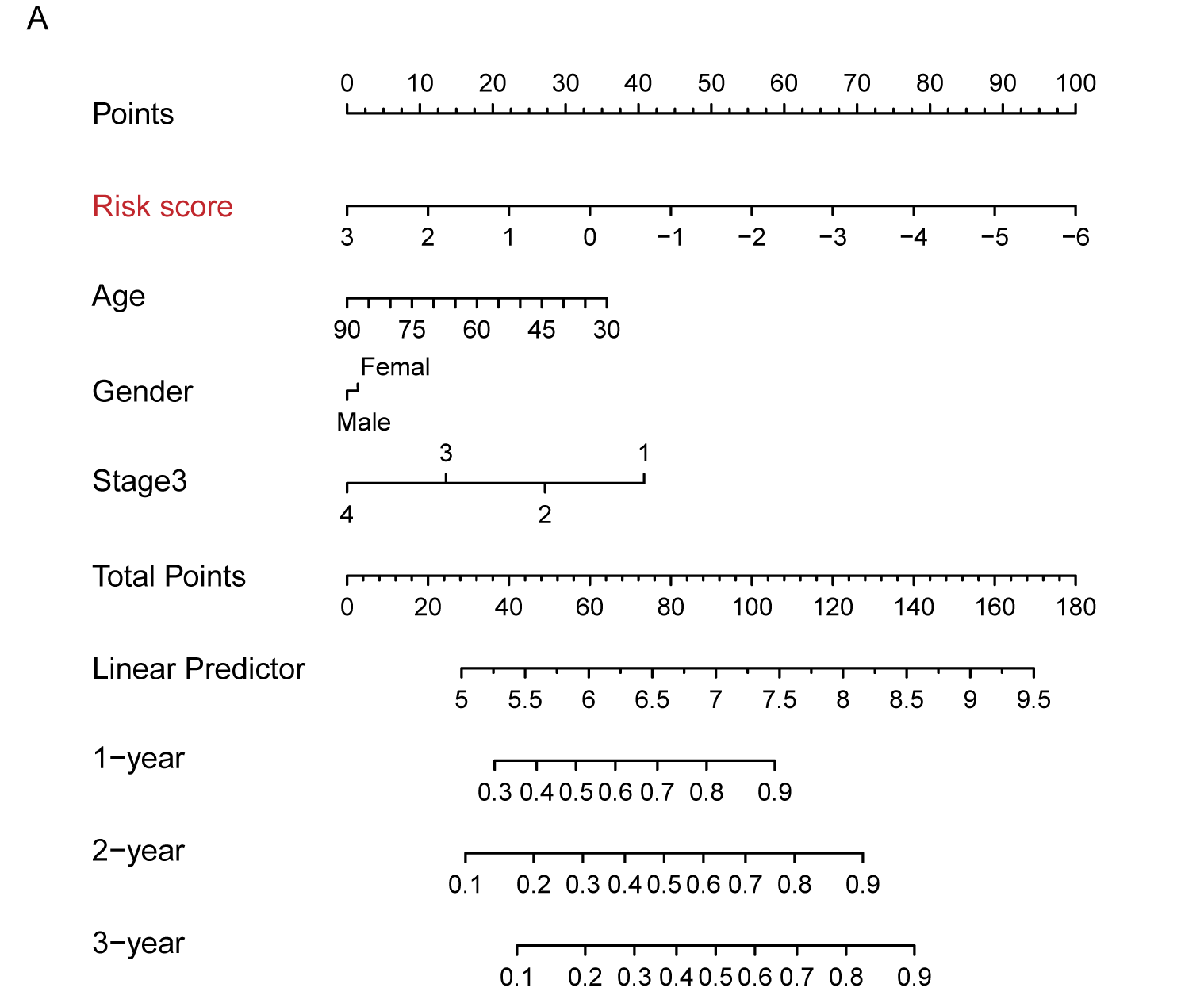

Supplement: Supplementary Figure 4 — A nomogram predicting the probability of the one-, two- and three-year overall survival. [file Image_4.tif]

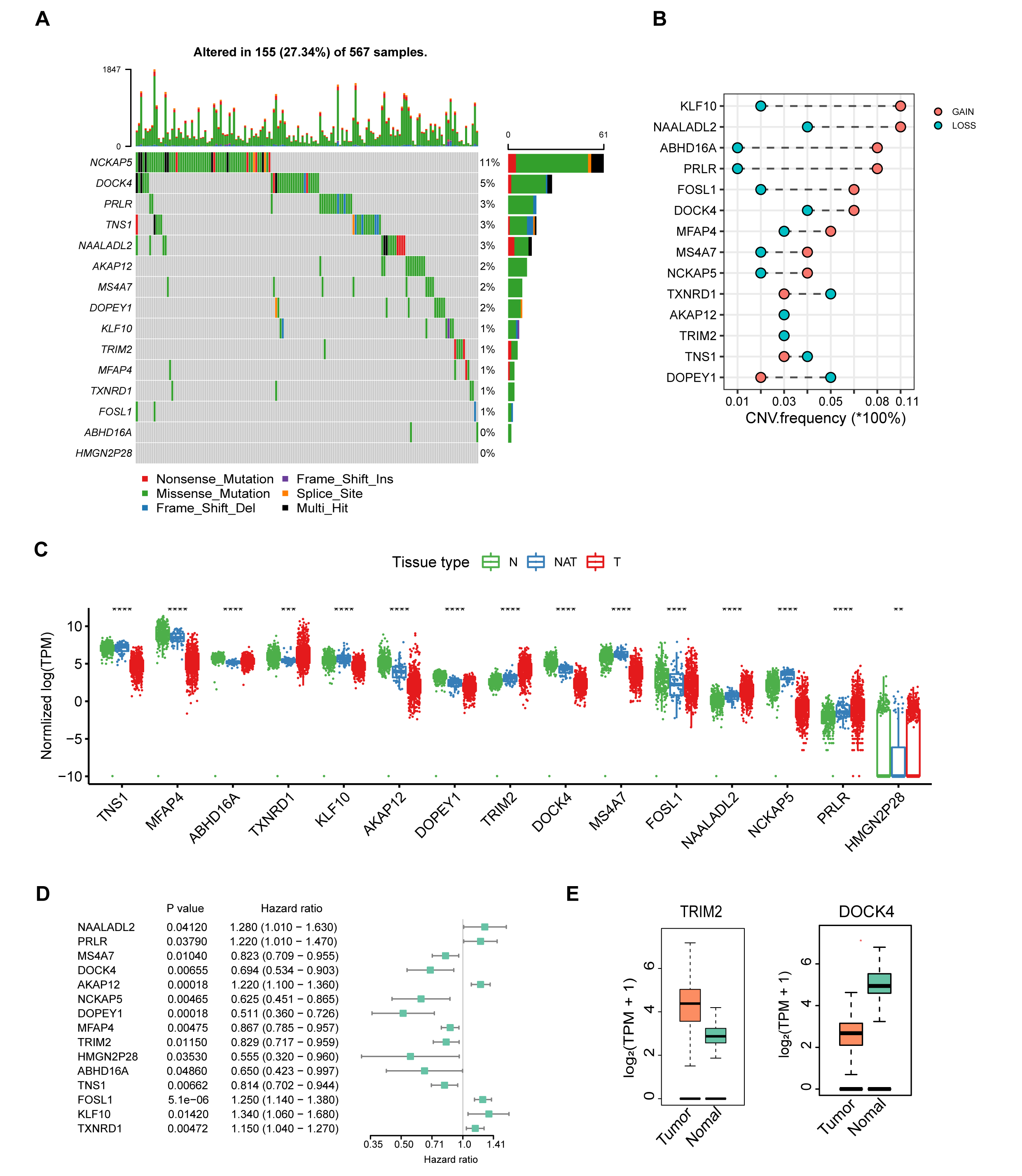

Supplement: Supplementary Figure 5 — The landscape of genetic and expression alterations of m6A-PPS. (A) A total of 155 of 567 LUAD patients experienced genetic alterations of 15 m6A-PPS genes. The number on the right indicates the mutation frequency in each gene, and each column represents every individual. (B) The CNV frequency of m6A-PPS was low. (C) Average expression of 15 m6A-PPS genes in normal tissues from the GTEx database, tumor tissues and paired normal tissues from the TCGA-LUAD cohort. Asterisks show the level of significance among different tissue types based on the Kruskal–Wallis test (**P< 0.01, *** P< 0.001, ****P< 0.0001). (D) Forest plot of the prognostic ability of 15 m6A-regulated genes using the multivariate Cox proportional hazard model. (E) The mRNA expression levels of TRIM2 and DOCK4 in 483 tumors and 347 normal tissues from the GEPIA2 database. [file Image_5.tif]

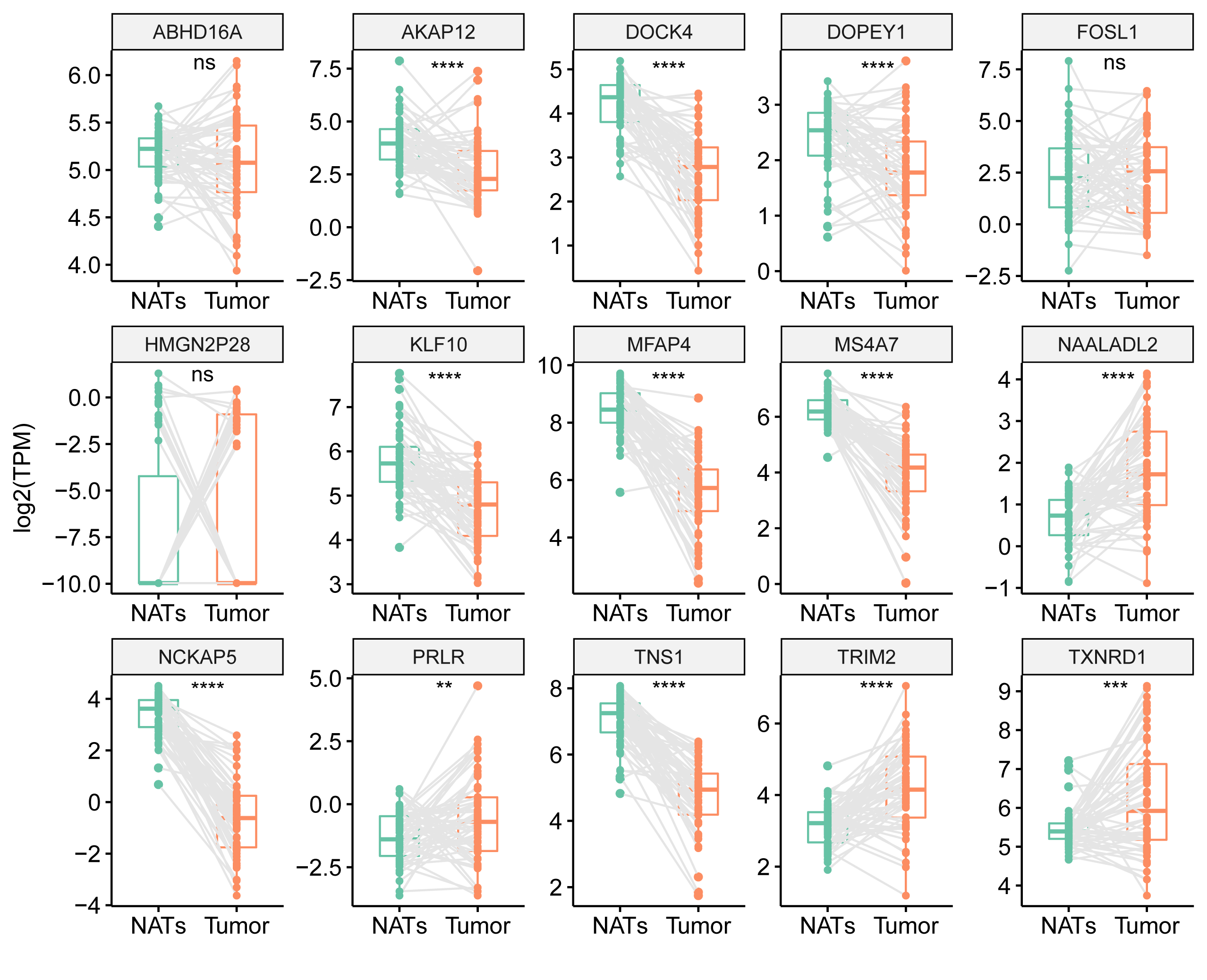

Supplement: Supplementary Figure 6 — The expression overview of 15 m6A-PPS genes between tumor and paired normal tissues. (ns P>0.05, **P< 0.01, *** P< 0.001, ****P< 0.0001). [file Image_6.tif]
